# Supplementary material for: GTPBP8 plays a role in mitoribosome formation in human mitochondria
Source: Nat Commun. 2024 Jul 5;15:5664. doi: 10.1038/s41467-024-50011-x (PMC11229512; doi:10.1038/s41467-024-50011-x)
Supplement: Supplementary file 1 — Supplementary Information [file 41467_2024_50011_MOESM1_ESM.pdf]

SUPPLEMENTARY INFORMATION

GTPBP8 plays a role in mitoribosome formation in human mitochondria

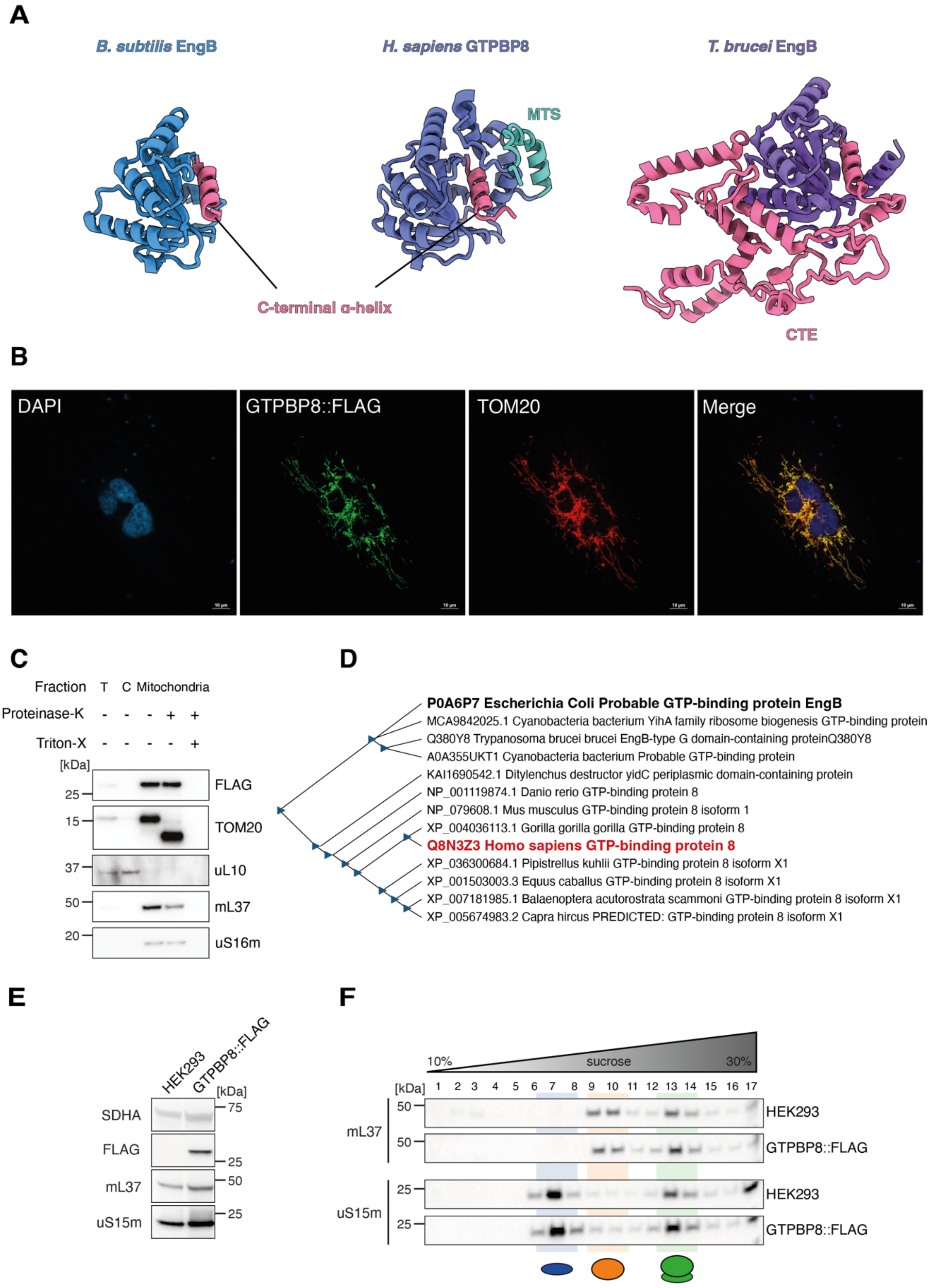

Supplementary Fig. 1. GTPBP8 localizes in mitochondria in mammalian cells.

(A) Comparison of 3D structures between *H. sapiens* GTPBP8 (middle), *B. subtilis* EngB (left), and *T. brucei* EngB (right). The predicted structure of human GTPBP8 was retrieved from the AlphaFold Protein Structure Database [1,2] and compared to *B. subtilis* EngB crystal structure [3] and *T. brucei* cryo-EM structure (PDB: 6YXY, [4]). Bacterial EngB and human GTPBP8 C-terminal

helices are highlighted in pink as well as *T. brucei* EngB CTE. GTPBP8 mitochondrial targeting sequence (MTS) is colored light green.

(B) HOS cells were transiently transfected with GTPBP8::FLAG cDNA. DAPI (blue) was used to label the cell nuclei. An anti-FLAG antibody was used to identify the GTPBP8::FLAG protein product, and anti-mouse secondary antibody conjugated to Alexa Fluor 488 (green) was used to visualize it. Anti-TOM20 antibody, an integral outer mitochondrial membrane protein, and anti-rabbit secondary antibody coupled to AlexaFluor 568 (red) were used to detect the mitochondrial network. A digitally merged image of DAPI, GTPBP8::FLAG, and TOM20 signals reveals the colocalization of GTPBP8::FLAG within mitochondria.

(C) Subcellular fractionation of GTPBP8::FLAG overexpressing cells. Cell lysates were separated into cytosolic and mitochondrial fractions. The mitochondrial fractions were either untreated or treated with 25 µg/ml Proteinase K with or without the addition of 1% Triton X-100. Fractions were further analyzed via western blotting: anti-FLAG antibody was used to detect GTPBP8::FLAG, anti-uL10 (cytosolic ribosomal protein uL10) antibody was used as a marker of the cytosol, anti-TOM20 antibody was used as a marker of the outer mitochondrial membrane, anti-mL37 and anti-uS16m antibodies were used as markers of the mitochondrial matrix. T, total cell lysate; C, cytosol.

(D) Phylogenetic analysis of human GTPBP8. The tree was generated using the Geneious software.

(E) MRPs steady-state levels in HEK293 overexpressing GTPBP8::FLAG. Mitochondrial lysates from HEK293 and GTPBP8::FLAG overexpressing cells were analyzed via western blotting. Cells were induced with 50 ng/ml of doxycycline prior to the experiment. Membranes were probed with anti-FLAG antibodies to assess GTPBP8 overexpression, anti-mL37 and anti-uS15m antibodies. Anti-SDHA antibody was used as the loading control.

(F) Sucrose gradient centrifugation analysis to assess mitoribosome sedimentation patterns in GTPBP8::FLAG overexpressing cells compared to HEK293 control cells. Mitochondrial lysates were loaded onto 10-30 % isokinetic sucrose gradients and obtained fractions were analyzed via western blotting. Membranes were probed for mt-LSU MRP mL37, mt-SSU MRP uS15m and GTPBP8. Source data are provided as a Source Data file.

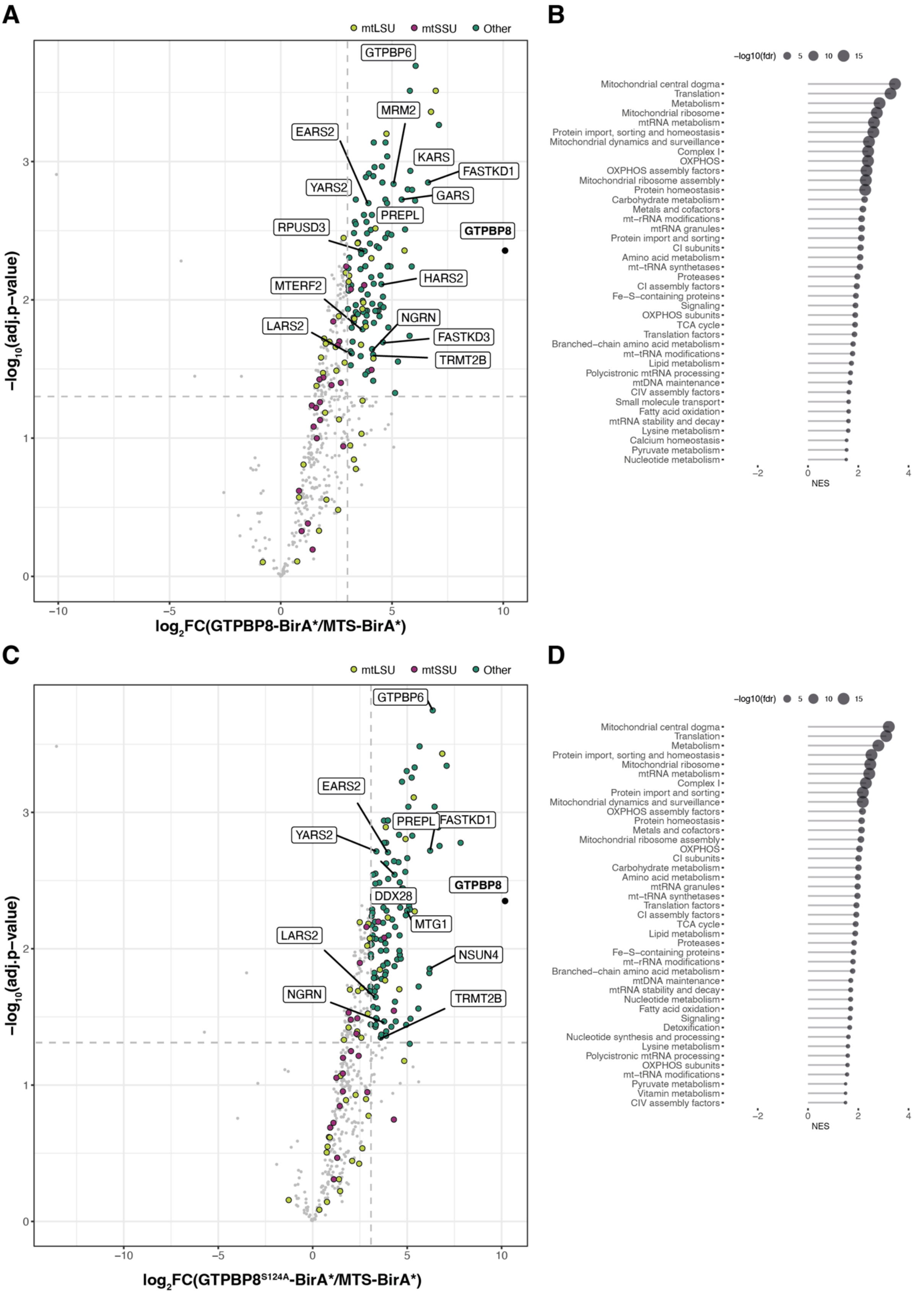

Supplementary Fig. 2. GTPBP8 BioID analysis.

(A) Label-free quantitative (LFQ) mass spectrometry analysis following GTPBP8-BioID experiment (n = 3 independent experiments). Mitochondrial targeted BirA* (MTS-BirA*) was

used as a control. The $\log_2$(fold change) and the $-\log_{10}$(adjusted p-value) are displayed on the *x*- and *y*- axis respectively. The light green dots highlight mt-LSU MRPs, whereas the purple dots highlight mt-SSU MRPs. Other proteins are highlighted in turquoise. The dashed lines (light grey) represent the thresholds of the $\log_2 FC > 3$ and $-\log_{10}$(adj.p-value) > 1.3 (adj.p-value < 0.05). The volcano plot was filtered for mitochondrial proteins as annotated in MitoCarta 3.0. A full annotation of the identified proteins is displayed in Supplementary Data 2.

(B) Mitocarta3 pathway enrichment analysis performed using WebGestalt of GTPBP8-BioID results. The $-\log_{10}$ of the false discovery rate (FDR) and the normalized enrichment score (NES) are indicated.

(C) Label-free quantitative (LFQ) mass spectrometry analysis following GTPBP8^{S124A}-BioID experiment ($n = 3$ independent experiments). Mitochondrial targeted BirA* (MTS-BirA*) was used as a control. The $\log_2$(fold change) and the $-\log_{10}$(adjusted p-value) are displayed on the *x*- and *y*- axis respectively. The light green dots highlight mt-LSU MRPs, whereas the purple dots highlight mt-SSU MRPs. Other proteins are highlighted in turquoise. The dashed lines (light grey) represent the thresholds of the $\log_2 FC > 3$ and $-\log_{10}$(adj.p-value) > 1.3 (adj.p-value < 0.05). The volcano plot was filtered for mitochondrial proteins as annotated in MitoCarta 3.0. A full annotation of the identified proteins is displayed in Supplementary Data 2.

(D) Mitocarta3 pathway enrichment analysis performed using WebGestalt of GTPBP8^{S124A}-BioID results. The $-\log_{10}$ of the false discovery rate (FDR) and the normalized enrichment score (NES) are indicated.

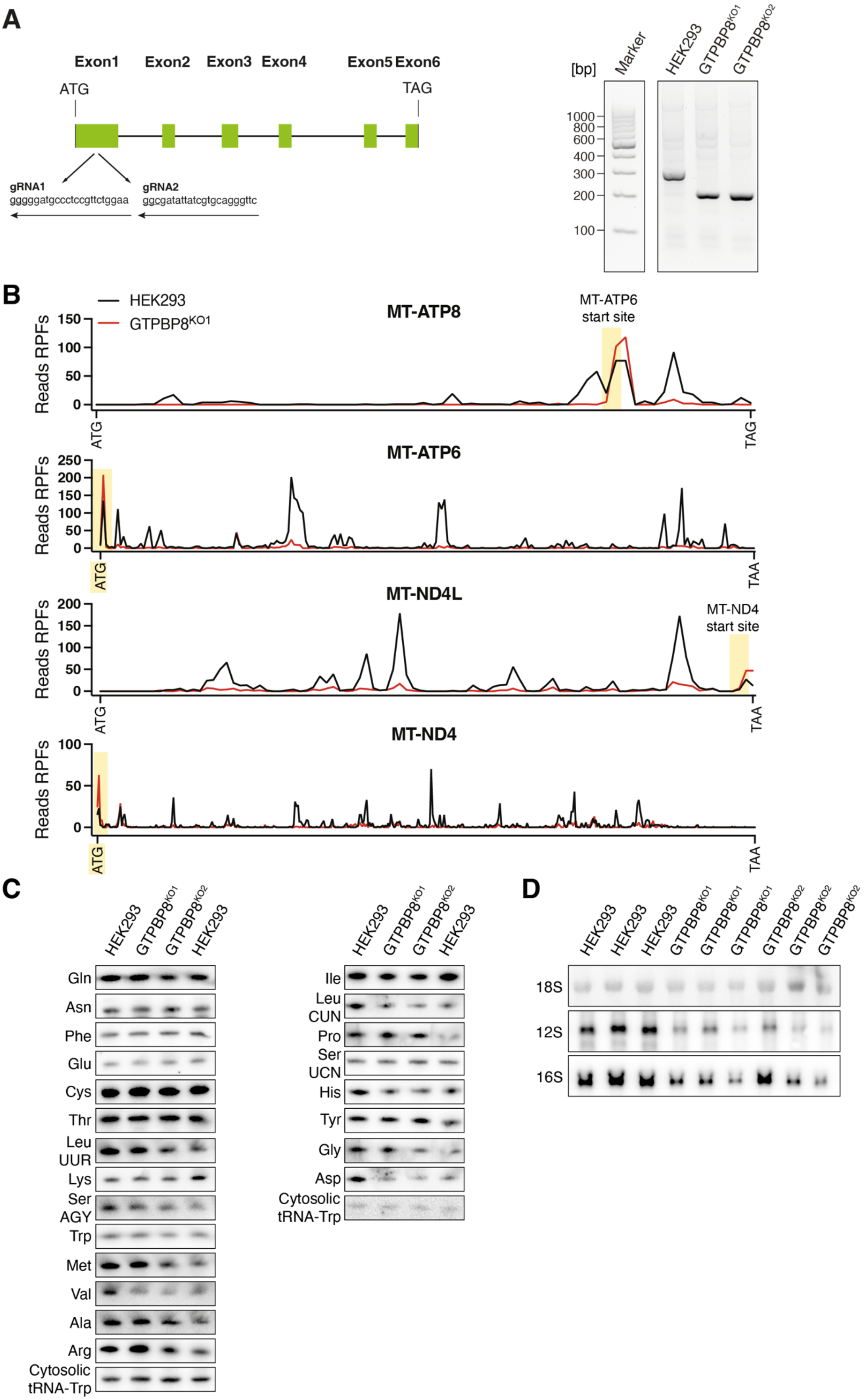
A
Exon1
Exon2
Exon3
Exon4
Exon5
Exon6
ATG
TAG
gRNA1
gggggatgccctccgttctggaa
gRNA2
ggcgatattatcgtgcagggttc
[bp]
Marker
HEK293
GTPBP8KO1
GTPBP8KO2
1000
800
600
400
300
200
100
B
HEK293
GTPBP8KO1
MT-ATP8
MT-ATP6 start site
Reads RPFs
150
100
50
0
ATG
TAG
MT-ATP6
250
200
150
100
50
0
ATG
TAA
MT-ND4L
MT-ND4 start site
200
150
100
50
0
ATG
TAA
MT-ND4
100
50
0
ATG
TAA
C
HEK293
GTPBP8KO1
GTPBP8KO2
HEK293
Gln
Asn
Phe
Glu
Cys
Thr
Leu UUR
Lys
Ser AGY
Trp
Met
Val
Ala
Arg
Cytosolic tRNA-Trp
Ile
Leu CUN
Pro
Ser UCN
His
Tyr
Gly
Asp
Cytosolic tRNA-Trp
D
HEK293
HEK293
HEK293
GTPBP8KO1
GTPBP8KO1
GTPBP8KO1
GTPBP8KO2
GTPBP8KO2
GTPBP8KO2
18S
12S
16S

Supplementary Fig. 3. Generation of GTPBP8 knock-out model and its effect on mitochondrial gene expression.

(A) CRISPR/Cas9 mediated targeted GTPBP8 knock-out. The schematic indicates the target sites of gRNAs within the protein-coding exon 1. Underlined sequences represent the PAM sites. On the right, PCR analysis was performed targeting *GTPBP8* exon 1 gRNAs cutting sites.

(B) Representation of the RPFs profiles on ATP8/ATP6 and ND4L/ND4 transcripts in GTPBP8^{KO1} (red) compared to HEK293 (black). The start (ATG) and the stop (TAA) codons are indicated.

(C) Northern blotting to assess steady-state levels of mt-tRNAs in GTPBP8^{KO1} and GTPBP8^{KO2} compared to HEK293. The cytosolic tRNA for tryptophan (tRNA-Trp) was used as a loading control.

(D) Northern blotting to assess steady-state levels of mt-rRNAs in GTPBP8^{KO1} and GTPBP8^{KO2} compared to HEK293. Nuclear-encoded 18S rRNA was used as a loading control. Source data are provided as a Source Data file.

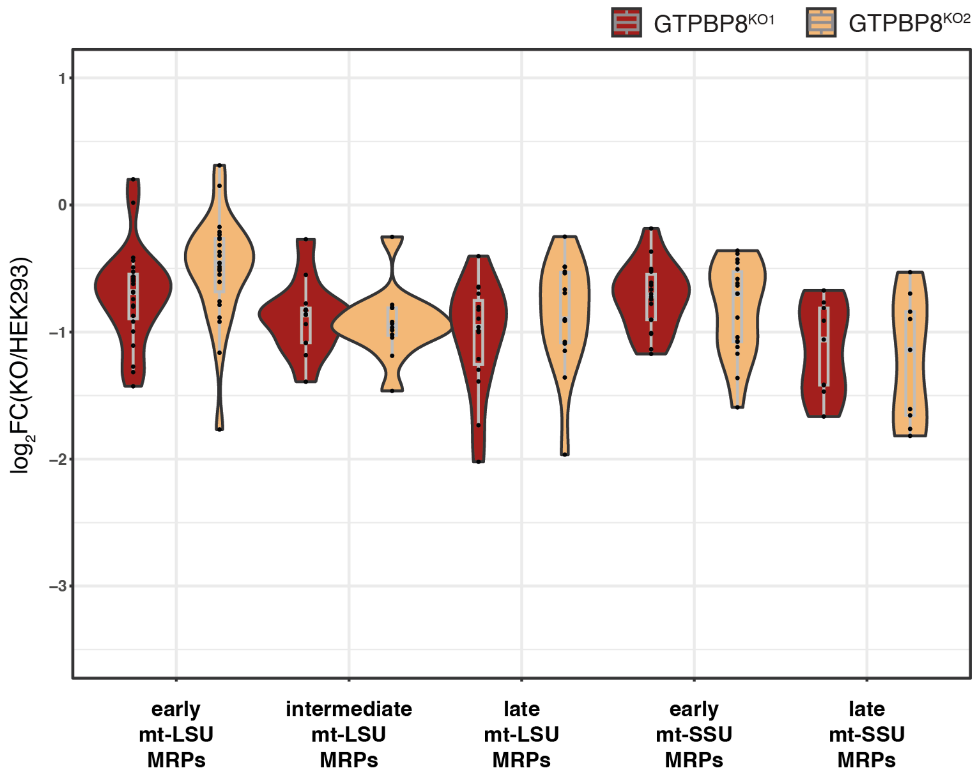

Supplementary Fig. 4. Effect of GTPBP8 loss on mitoribosome assembly.

LFQ mass spectrometry analyses of early, intermediate and late mt-LSU MRPs and early and late mt-SSU MRPs based on [5] in GTPBP8^{KO1} and GTPBP8^{KO2} cell lines compared to HEK293 (n = 3 independent experiments). Log_2(FC) of GTPBP8^{KO1} vs HEK293 (red) and GTPBP8^{KO2} vs HEK293 (yellow) is represented on the y-axis. Summary of violin plots: early mt-LSU KO1 (median = -0.6860, min = -1.427, max = 0.202, q1 = -0.89500, q3 = -0.54400); early mt-LSU KO2 (median = -0.4580, min = -1.163, max = 0.312, q1 = -0.68250, q3 = -0.26400); intermediate mt-LSU KO1 (median = -0.8620, min = -1.391, max = -0.27, q1 = -1.08475, q3 = -0.81100); intermediate mt-LSU KO2 (median = -0.9495, min = -1.463, max = -0.251, q1 = -1.02650, q3 = -0.80950); late mt-LSU KO1 (median = -0.9610, min = -2.021, max = -0.403, q1 = -1.25450, q3 = -0.75100); late mt-LSU KO2 (median = -0.9000, min = -1.965, max = -0.249, q1 = -1.08450, q3 = -0.53100); early mt-SSU KO1 (median = -0.7125, min = -1.172, max = -0.185, q1 = -0.90350, q3 = -0.54575); early mt-SSU KO2 (median = -0.6980, min = -1.593, max = -0.359, q1 = -1.07550, q3 = -0.52500); late mt-SSU KO1 (median = -1.0590, min = -1.665, max = -0.673, q1 = -1.41800, q3 = -0.81100); late mt-SSU KO2 (median = -1.1400, min = -1.818, max = -0.529, q1 = -1.65600, q3 = -0.84000). The complete list of log_2(FC) values is presented in Supplementary Data 3.

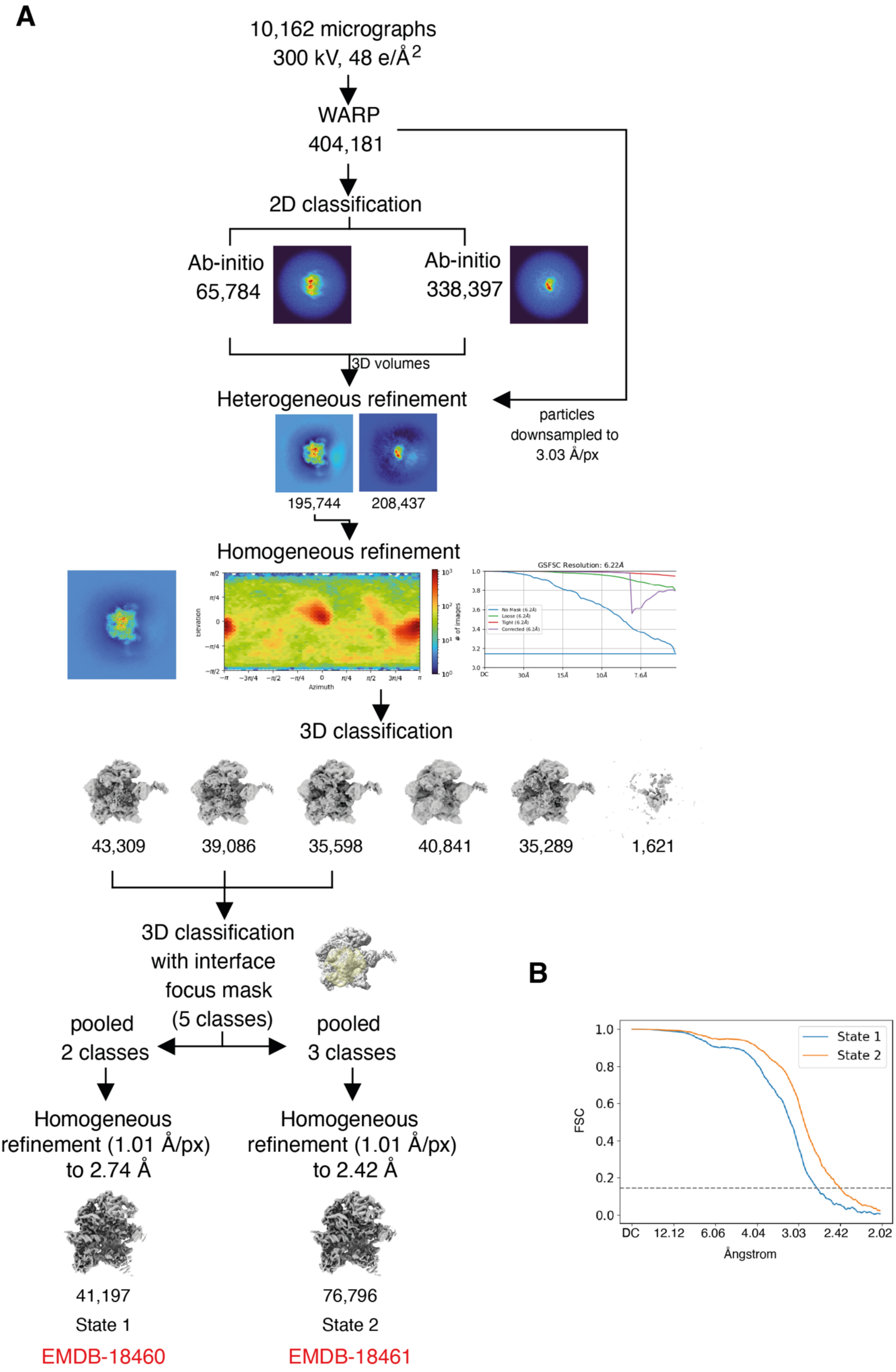

Supplementary Fig. 5. Data processing of the mt-LSU sample from GTPBP8^{KO1} cells.

(A) Data processing strategy for the mt-LSU assembly intermediates derived from $GTPBP8^{KO1}$ cells. The homogeneous reconstruction as well as State 1 and State 2 3D classes are deposited as EMDB-18460 and EMDB-18461, respectively. For the homogenous refinement, the 3D particle poses and the half-maps FSC from CryoSPARC v4 are shown.
(B) Gold-standard Fourier shell correlation (FSC) for the two EMDB-deposited reconstructions. The horizontal dashed line indicates the FSC cut-off at 0.143.

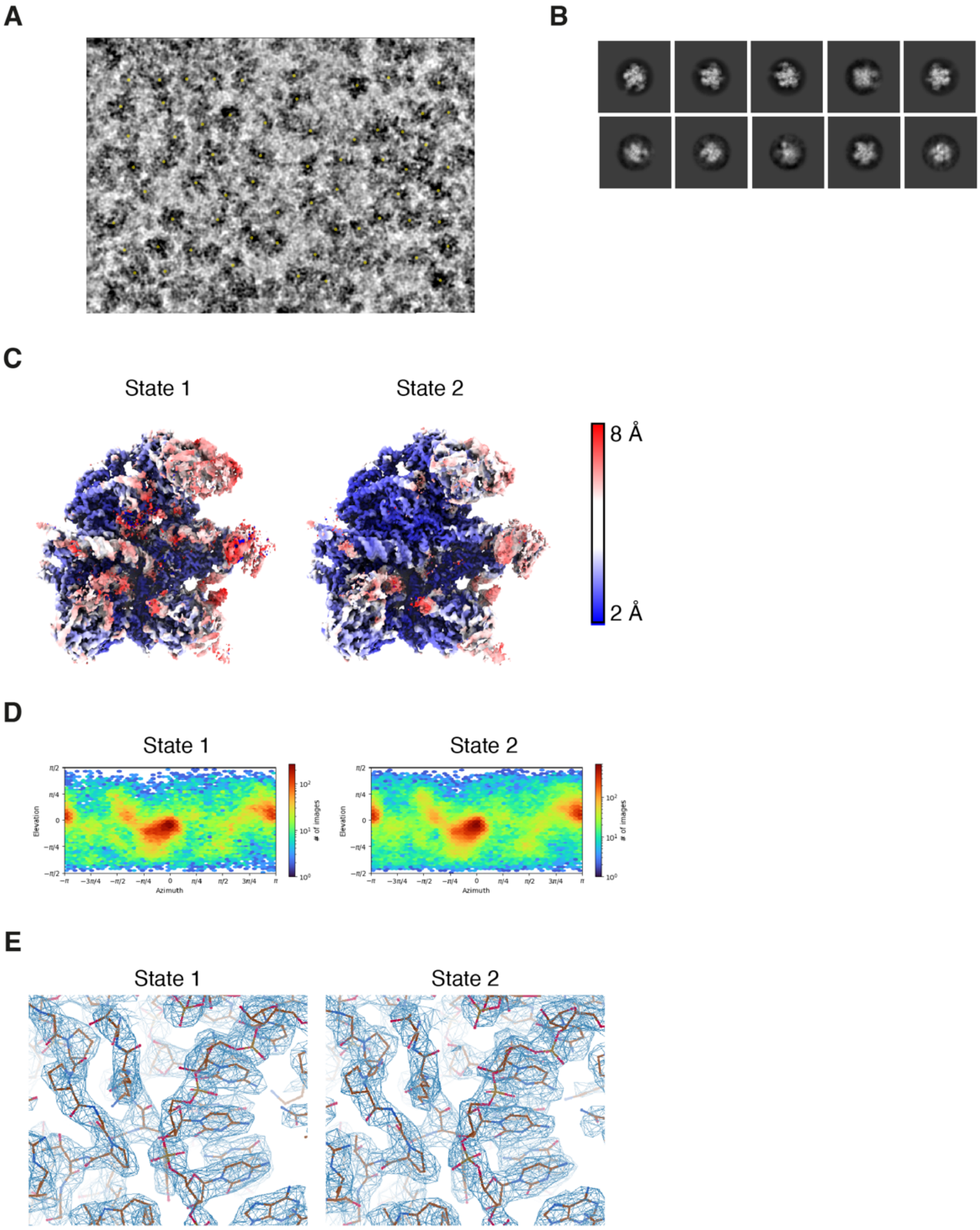

Supplementary Fig. 6. Data processing of the mt-LSU sample from GTPBP8^{KO1} cells.

(A) Representative micrograph for the mt-LSU dataset. The micrograph was deconvolved in WARP [5] for visualization. The picked particle coordinates are shown with yellow dots.

(B) Selected 2D classes.

(C) Local resolution estimation (FSC threshold 0.5) calculated by BlocRes [6] as implemented in cryoSPARC v4.3.0.

(D) Distribution of particle orientations (Obtained from CryoSPARC v4.3.0).

(E) Representative cryo-EM density (state 1, 5.4 RMSD; state 2, 4.5 RMSD).

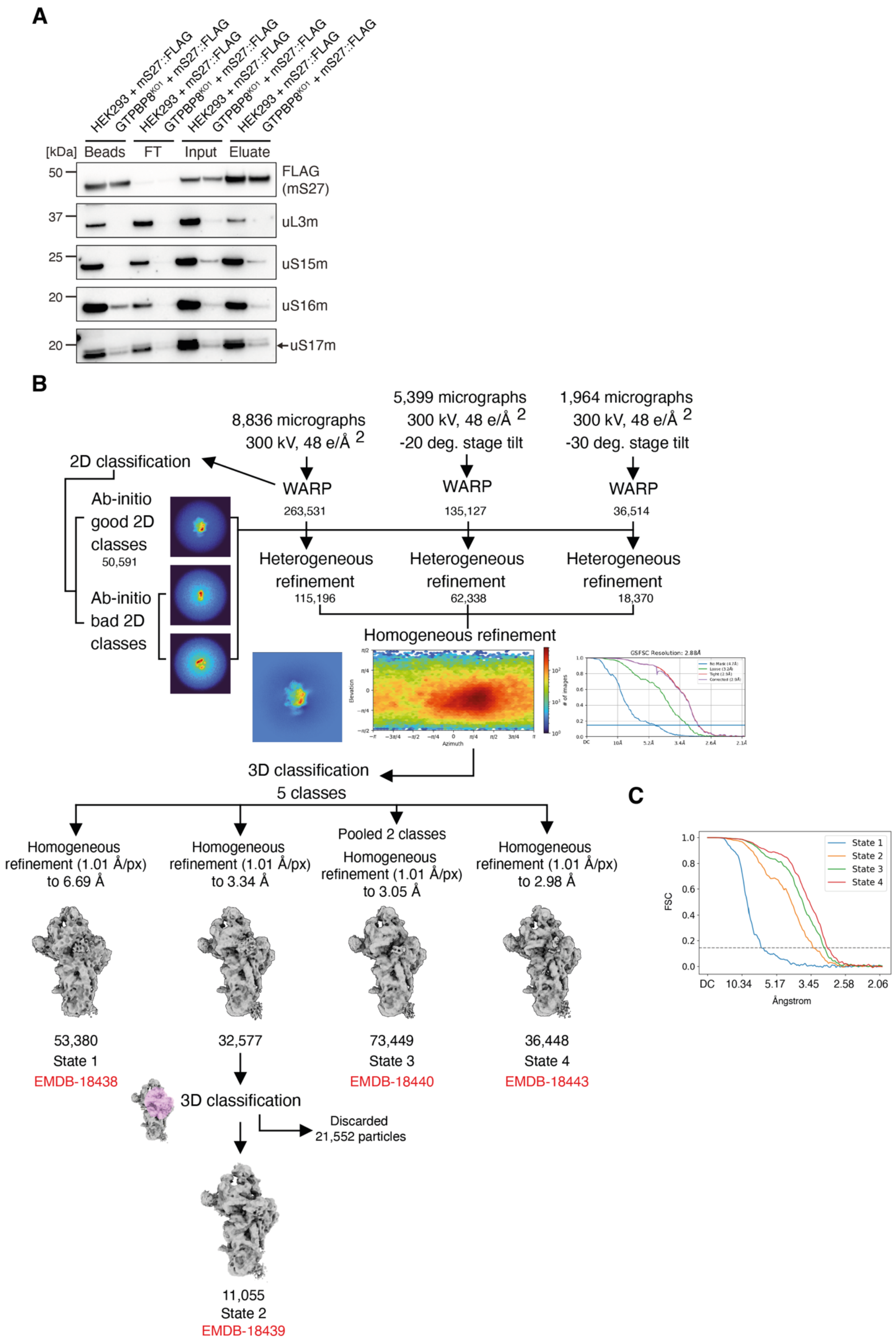

Supplementary Fig. 7. Data processing of the mt-SSU sample from GTPBP8^{KO1} cells.

(A) Immunoblotting of mS27::FLAG-IP from GTPBP8^{KO1} cells. Beads, flow-through (FT), mitochondrial lysates (Input) and eluates from HEK293 overexpressing mS27::FLAG and GTPBP8^{KO1} overexpressing mS27::FLAG were resolved via SDS-PAGE. Membranes were probed with antibodies against FLAG and for the mt-LSU MRP uL3m, and the mt-SSU MRPs uS15m, uS16m, and uS17m. Source data are provided as a Source Data file.

(B) Data processing strategy for the mt-SSU particles obtained by mS27 immunoprecipitation from GTPBP8^{KO1} cells. The homogeneous reconstruction as well as State 1, 2, 3 and 4 3D classes are deposited as EMDB-18438, EMDB-18439, EMDB-18440, EMDB-18443, respectively. For the homogenous refinement, the 3D particle poses and the half-maps FSC from CryoSPARC v4 are shown.

(C) Gold-standard Fourier shell correlation (FSC) for the four EMDB-deposited reconstructions. The horizontal dashed line indicates the FSC cut-off at 0.143.

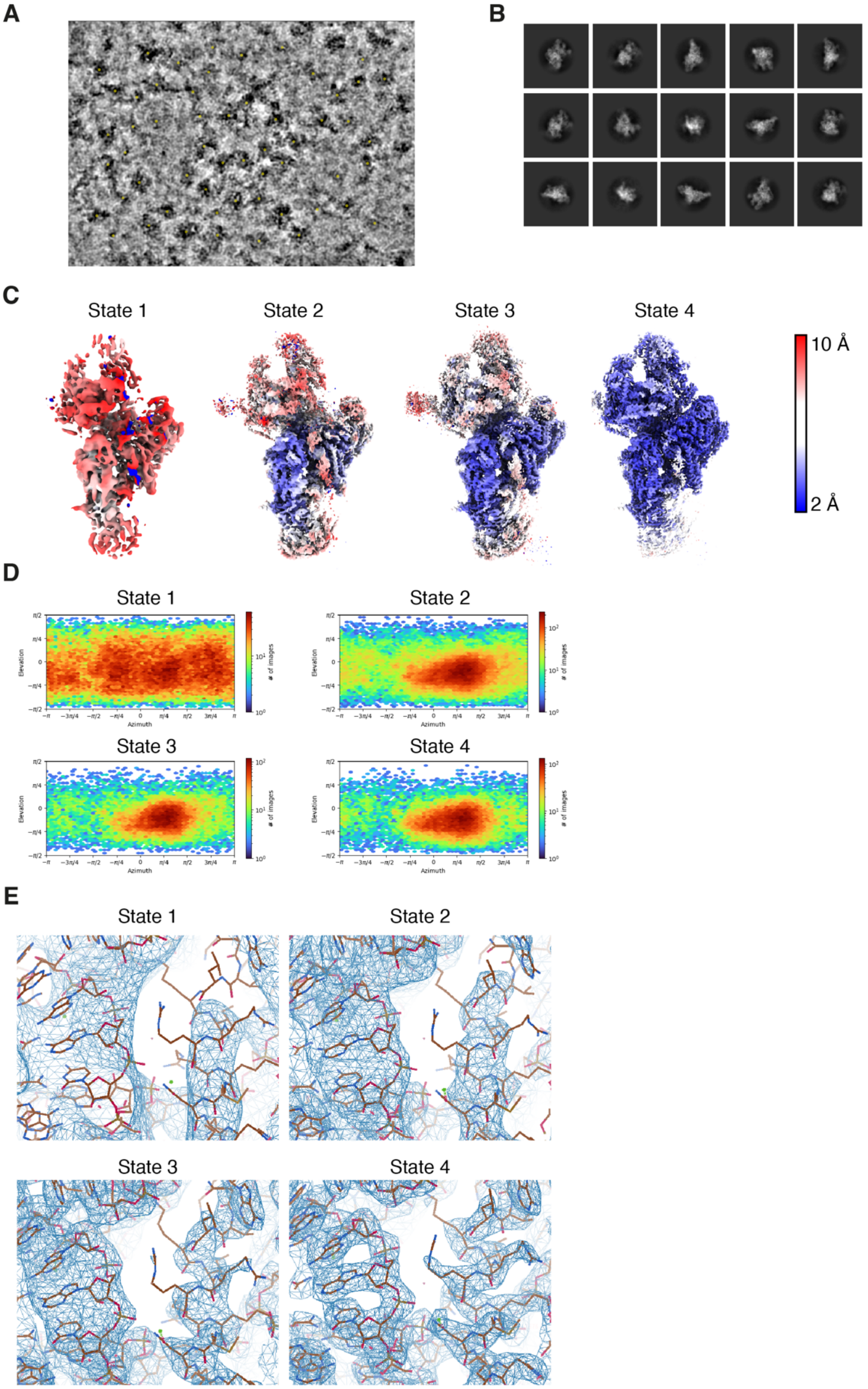

Supplementary Fig. 8. Data processing of the mt-SSU sample from GTPBP8^{KO1} cells.

(A) Representative micrograph for the mt-SSU dataset. The micrograph was deconvolved in WARP [6] for visualization. The picked particle coordinates are shown with yellow dots.

(B) Selected 2D classes.

(C) Local resolution estimation (FSC threshold 0.5) calculated by BlocRes [7] as implemented in cryoSPARC v4.3.0.

(D) Distribution of particle orientations (Obtained from CryoSPARC v4.3.0).

(E) Representative cryo-EM density (state 1, 4.5 RMSD; state 2, 4.8 RMSD; state 3, 5.6 RMSD; state 4, 4.9 RMSD).

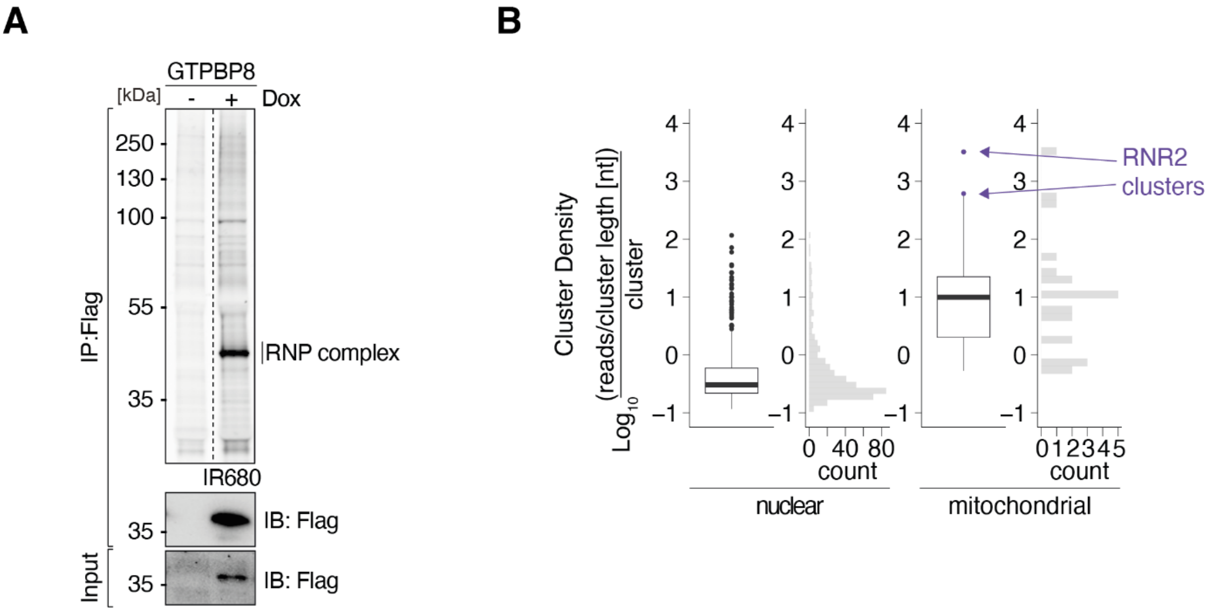

Supplementary Fig. 9. fPAR-CLIP analysis

(A) IR680 fluorescent image of crosslinked and fluorescent adapter-ligated GTPBP8::FLAG ribonucleoprotein complexes in HEK293 cells with or without doxycycline induction. GTPBP8::FLAG immunoblots depict the efficiency of the pulldown and doxycycline induction (n = 3 independent experiments). Source data are provided as a Source Data file.

(B) GTPBP8 RNA binding sites (coined clusters by standard fPAR-CLIP analysis) were parsed by nuclear and mitochondrial encoded transcripts. Reads per cluster median and distribution is presented (T-test p-value=1.74×10^{-6}), as well as the orthogonal histogram of the prevalence of cluster per cluster intensity (reads/cluster). Clusters along the 16S mt-rRNA (RNR2) are colored in purple.

Supplementary Table 1. List of primers used for gene cloning

Primer Sequence (5'→3')	
BamHI_GTPBP8_Fwd (pcDNA5; FLAG-IP)	CTTTCTTGGATCCAAGGGAGAATGGCGGCGCC
GTPBP8_FLAG_XhoI_Rev (pcDNA5; FLAG-IP)	CTTTCTTTCTCGAGCTACTTATCGTCGTCATCCTT GTAATCGTCAAGACTTCCTGTTACACTG
GTPBP8_S124A_Fwd	ATGTTGGAAAAGCATCTCTAATCAAG
GTPBP8_S124A_Rev	CTTGATTAGAGATGCTTTTCCAACATT
BamHI_mS27_Fwd	GAATCGTCTCtGATCGCCGCCACCATGGCTGCCTC CATAGTGCG
mS27_FLAG_XhoI_Rev	GAATCGTCTCtTCGACTACTTATCGTCGTCATCCTT GTAATCGGCAGATGCCTTTGCTGCTT
NheI_GTPBP8_Fwd (pcDNA3; BioID)	CACAGCTAGCATGGCGGCGCCCGGGCTG
GTPBP8_BamHI_Rev (pcDNA3; BioID)	CACAGGATCCGTCAAGACTTCCTGTTACACTGGC
AflII_GTPBP8_Fwd (pcDNA5; BioID)	CACACTTAAGATGGCGGCGCCCGGGC
GTPBP8_NotI_Rev (pcDNA5; BioID)	CACAGCGGCCGCCTATGCGTAATCCGGTAC

Supplementary Table 2. List of Antibodies

**Antibody**	**Company**	**Catalog No.**
Anti-FLAG	Abcam	ab1257
GTPBP8	Prestige Antibodies, Sigma-Aldrich	HPA034831
GAPDH	Abcam	ab8245
SDHA	Abcam	ab14715
MRPL3/uL3m	Prestige Antibodies, Sigma-Aldrich	HPA043665
MRPL37/mL37	Prestige Antibodies, Sigma-Aldrich	HPA025826
MRPL12/bL12m	Prestige Antibodies, Sigma-Aldrich	HPA022853

MRPL28/bL28m	Prestige Antibodies, Sigma-Aldrich	HPA030594
MRPL49/mL49	Proteintech Group	15542-1-AP
MRPL15/uL15m	Proteintech Group	18339-1-AP
MRPS16/uS16m	Prestige Antibodies, Sigma-Aldrich	HPA054538
MRPS37/mS37 (CHCHD1)	Invitrogen	PA-58635
MRPS15/uS15m	Proteintech Group	17006-1
MRPS17/uS17m	Proteintech Group	18881-1
MRPS22/mS22	Thermo Fisher	10984-1-AP
RPLP0/uL10	Invitrogen	PA5-89335
OXPHOS human WB	Abcam	ab110411
TOM20	Santa-Cruz	sc-11415
MTG1	Prestige Antibodies, Sigma-Aldrich	HPA037827
MRM3/RNMTL1	Prestige Antibodies, Sigma-Aldrich	HPA023292
RBFA	Abcam	ab224741
GTPBP10	Prestige Antibodies, Sigma-Aldrich	HPA021076
TRMT10C	Prestige Antibodies, Sigma-Aldrich	HPA036671
HRP secondary rabbit	GE Healthcare	NA9340V
HRP secondary mouse	GE Healthcare	NA9310V
HRP secondary goat	Santa-Cruz	sc-2354

Supplementary Table 3. List of primers for rRNAs and tRNAs

**Primer Sequence (5’→3’)**	
12S_Fwd	CACTGAAAATGTTTAGACGGG
12S_Rev	GGCTCCTCTAGAGGGATATG
16S_Fwd	TAGATATAGTACCGCAAGGG
16S_Rev	GACTTGTTGGTTGATTGTA

H_T7_Met_68_Fwd	TAATACGACTCACTATAGGGAGACtagtacgggaagggtataacc
H_Met_68_Rev	agtaaggtcagctaaataagctatc
H_T7_Val_69_Fwd	TAATACGACTCACTATAGGGAGACtcagagcggtcaagttaagttg
H_Val_69_Rev	cagagtgtagcttaacacaaagc
L_T7_Gln_72_Fwd	TAATACGACTCACTATAGGGAGACctaggactatgagaatcgaac
L_Gln_72_Rev	taggatggggtgtgatagg
L_T7_Tyr_66_Fwd	TAATACGACTCACTATAGGGAGACtggtaaaaagaggcctaaccc
L_Tyr_66_Rev	ggtaaaatggctgagtgaagc
L_T7_Pro_68_Fwd	TAATACGACTCACTATAGGGAGACtcagagaaaaagtctttaactc
L_Pro_68_Rev	cagagaatagtttaaattagaatc
L_T7_Glu_69_Fwd	TAATACGACTCACTATAGGGAGACtattctcgcacggactacaac
L_Glu_69_Rev	gttcttgtagttgaaatacaacg
L_T7SerUCN_69_Fwd	TAATACGACTCACTATAGGGAGACcaaaaaggaaggaatcgaacc
L_SerUCN_69_Rev	gaaaaagtcatggaggccatg
L_T7_Cys_66_Fwd	TAATACGACTCACTATAGGGAGACaagccccggcaggtttgaag
L_Cys_66_Rev	agctccgaggtgattttcatattg
L_T7_Asn_73_Fwd	TAATACGACTCACTATAGGGAGACctagaccaatgggacttaaac
L_Asn_73_Rev	tagattgaagccagttgattag
L_T7_Ala_69_Fwd	TAATACGACTCACTATAGGGAGACtaaggactgcaaaaccccac
L_Ala_69_Rev	aagggcttagcttaattaaagtgg
H_T7_Phe_71_Fwd	TAATACGACTCACTATAGGGAGACtgtttatggggtgatgtgag
H_Phe_71_Rev	gtttatgtagcttacctcctc
H_T7LeuUUR_75_Fwd	TAATACGACTCACTATAGGGAGACtgttaagaagaggaattgaacctctg
H_LeuUUR_75_Rev	gttaagatggcagagcccgg
H_T7_Ile_69_Fwd	TAATACGACTCACTATAGGGAGACtagaaataagggggtttaagctc
H_Ile_69_Rev	agaaatatgtctgataaaagagttac
H_T7_Trp_68_Fwd	TAATACGACTCACTATAGGGAGACcagaaattaagtattgcaacttac
H_Trp_68_Rev	agaaatttaggttaaatacagacc
H_T7_Asp_68_Fwd	TAATACGACTCACTATAGGGAGACtaagatatataggatttagcctata
H_Asp_68_Rev	aaggtattagaaaaaccatttcat

H_T7_Lys_70_Fwd	TAATACGACTCACTATAGGGAGACtcactgtaaagaggtgttgg
H_Lys_70_Rev	cactgtaaagctaacttagcat
H_T7_Gly_68_Fwd	TAATACGACTCACTATAGGGAGACtactctttttgaatgttgtcaaaac
H_Gly_68_Rev	actcttttagtataaatagtaccg
H_T7_Arg_65_Fwd	TAATACGACTCACTATAGGGAGACttggtaaatatgattatcataatttaatg
H_Arg_65_Rev	tggtatatagtttaaacaaaacgaa
H_T7_His_69_Fwd	TAATACGACTCACTATAGGGAGACggtaaataaggggtcgtaagc
H_His_69_Rev	gtaaatatagtttaaccaaaacatcag
H_T7SerAGY_59_Fwd	TAATACGACTCACTATAGGGAGACtgagaaagccatgttgttagac
H_SerAGY_59_Rev	gagaaagctcacaagaactg
H_T7LeuCUN_71_Fwd	TAATACGACTCACTATAGGGAGACtacttttatttggagttgcacc
H_LeuCUN_71_Rev	acttttaaaggataacagctatcc
H_T7_Thr_66_Fwd	TAATACGACTCACTATAGGGAGACtgtccttggaaaaaggttttcatc
H_Thr_66_Rev	gtccttgtagtataaactaatacac

Supplementary Table 4. List of TaqMan probes (Invitrogen)

**TaqMan probe Assay ID**	
ACTB	Hs99999903
MT-ND1	Hs02596873
MT-ND2	Hs02596874
MT-ND3	Hs02596875
MT-ND4	Hs02596876
MT-ND5	Hs02596878
MT-ND6	Hs02596879
MT-CYTB	Hs02596867
MT-COX1	Hs02596864
MT-COX2	Hs02596865
MT-COX3	Hs02596866
MT-ATP6	Hs02596862

MT-ATP8	Hs02596863
MT-RNR1	Hs02596859
MT-RNR2	Hs02596860

Supplementary Table 5. fPAR-CLIP adaptor sequences and primers

**Oligo name**	**Sequence**
3’ adapter	5’-rAppNNTGACTGTGGAATTCTCGGGT(fl)GCCAAGG-(fl) (MDX-O-226-29.51-2xAF660, Multiplexdx)
5’ adapter	5’(aminolinker-)GTTCAGAGTTCTACAGTCCGACGATCrNrNrNrN (MDX-O-264, Multiplexdx)
RT Primer	GCCTTGGCACCCGAGAATTCCA
5’ short PCR primer	CTTCAGAGTTCTACAGTCCGACGA
5’ long PCR primer	AATGATACGGCGACCACCGAGATCTACACGTTCAGAGTTCTACAGTCCGA
GTPBP8 Dox1 3’ Index primer	CAAGCAGAAGACGGCATACGAGATACGGTCTTGTGACTGGAGTTCCTTGGCACCCGAGAATTCCA
GTPBP8 Dox2 3’ Index primer	CAAGCAGAAGACGGCATACGAGATTCTCGCAAGTGACTGGAGTTCCTTGGCACCCGAGAATTCCA
GTPBP8 Dox3 3’ Index primer	CAAGCAGAAGACGGCATACGAGATGGAATTGCGTGACTGGAGTTCCTTGGCACCCGAGAATTCCA
GTPBP8 NoDox1 3’ Index primer	CAAGCAGAAGACGGCATACGAGATCTTCACCAGTGACTGGAGTTCCTTGGCACCCGAGAATTCCA

Supplementary Table 6. Cryo-EM data collection, refinement and validation statistics

	mt-SSU				mt-LSU	
	State 1 (EMDB-18438) (PDB-8QRK)	State 2 (EMDB-18439) (PDB-8QRL)	State 3 (EMDB-18440) (PDB-8QRM)	State 4 (EMDB-18443) (PDB-8QRN)	State 1 (EMDB-18460) (PDB-8QU1)	State 2 (EMDB-18461) (PDB-8QU5)
**Data collection and processing**						
Magnification	165,000	165,000	165,000	165,000	165,000	165,000
Voltage (kV)	300	300	300	300	300	300
Electron exposure (e–/Å^2)	48	48	48	48	48	48
Defocus range (μm)	0.2-2	0.2-2	0.2-2	0.2-2	0.2-2	0.2-2
Pixel size (Å)	1.01	1.01	1.01	1.01	1.01	1.01
Symmetry imposed	P1	P1	P1	P1	P1	P1
Initial particle images (no.)	195,904	195,904	195,904	195,904	404,181	404,181
Final particle images (no.)	53,380	11,055	73,499	36,448	41,197	76,769
Map resolution (Å)	6.69	3.96	3.05	2.98	2.74	2.42
FSC threshold	0.143	0.143	0.143	0.143	0.143	0.143
Map resolution range (Å)[1]						
Min	2.103	2.144	2.623	2.563	2.204	2.229
25th percentile	7.966	6.961	4.816	3.825	3.905	3.245
Median	9.034	9.070	6.878	6.264	6.733	6.009
75th percentile	10.768	11.309	8.575	8.529	8.790	7.972
Max	56.458	68.589	53.385	48.289	48.306	42.956
**Refinement**						
Initial model used (PDB code)	6RW4	7PNX	6RW4	7PO2	5OOM	5OOM
Model resolution (Å)	6.69	3.34	3.05	2.9	2.74	2.42
FSC threshold	0.143	0.143	0.143	0.143	0.143	0.143
Map sharpening *B* factor (Å^2)	351.0	30.1	40.2	39.4	25.6	27.2
Model composition						
Non-hydrogen atoms	66293	70955	68786	73195	85686	89817
Protein residues	5600	5932	5910	6287	7881	8105
Ligands: GNP/ZN/NAD/K/MG/SPM/ATP/SRY/FES/PNS/5FO/UNK	1/1/1/17/60/1/1/1/2/0/0/0	0/1/1/16/64/0/1/0/2/0/1/0	1/1/1/17/60/1/1/1/2/0/0/0	1/1/1/18/58/1/1/1/1/0/0/0	0/2/0/0/30/0/0/0/0/1/0/28	0/2/0/0/48/0/0/0/0/1/0/28
B factors (Å^2)						
Protein	0.00/953.69/287.42	7.39/66.45/27.03	0.00/973.01/110.90	0.00/212.14/71.23	9.60/999.00/150.77	12.00/837.35/116.52
Ligand	46.06/483.76/367.74	8.88/45.41/20.21	28.27/379.00/95.70	14.48/197.31/65.91	8.49/999.00/779.45	14.58/681.50/478.93
R.m.s. deviations						
Bond lengths (Å)	0.008 (36)	0.004 (22)	0.008 (36)	0.008 (43)	0.008 (3)	0.008 (6)
Bond angles (°)	0.872 (27)	0.797 (12)	0.885 (26)	0.906 (26)	1.211 (65)	1.464
Validation						
MolProbity score	1.27	0.95	1.33	1.27	2.33	2.00
Clashscore	3.05	1.86	3.59	3.11	12.22	7.14
Poor rotamers (%)	0.08	0.04	0.10	0.11	3.55	1.99
Ramachandran plot						
Favored (%)	96.98	98.82	96.97	97.04	95.56	94.44
Allowed (%)	2.98	3.04	2.98	2.91	4.40	5.48
Disallowed (%)	0.04	0.05	0.05	0.05	0.05	0.09

[1] Values reported by CryoSPARC Local Resolution Estimation inside the refinement mask.

Supplementary References

1. Jumper, J. *et al.* Highly accurate protein structure prediction with AlphaFold. *Nature* **596**, 583–589 (2021).

2. Varadi, M. *et al.* AlphaFold Protein Structure Database: massively expanding the structural coverage of protein-sequence space with high-accuracy models. *Nucleic Acids Res.* **50**, D439–D444 (2022).

3. Ruzheinikov, S. N. *et al.* Analysis of the Open and Closed Conformations of the GTP-binding Protein YsxC from Bacillus subtilis. *J. Mol. Biol.* **339**, 265–278 (2004).

4. Jaskolowski, M. *et al.* Structural Insights into the Mechanism of Mitoribosomal Large Subunit Biogenesis. *Mol. Cell* **79**, 629-644.e4 (2020).

5. Bogenhagen, D. F., Ostermeyer-Fay, A. G., Haley, J. D. & Garcia-Diaz, M. Kinetics and Mechanism of Mammalian Mitochondrial Ribosome Assembly. *Cell Rep.* **22**, 1935–1944 (2018).

6. Tegunov, D. & Cramer, P. Real-time cryo-electron microscopy data preprocessing with Warp. *Nat. Methods* **16**, 1146–1152 (2019).

7. Dai, M., Dong, Z., Xu, K. & Zhang, Q. C. CryoRes: Local Resolution Estimation of Cryo-EM Density Maps by Deep Learning. *J. Mol. Biol.* **435**, 168059 (2023).
