# Supplementary material for: GTPBP8 plays a role in mitoribosome formation in human mitochondria
Source: Nat Commun. 2024 Jul 5;15:5664. doi: 10.1038/s41467-024-50011-x (PMC11229512; doi:10.1038/s41467-024-50011-x)
Supplement: Supplementary file 3 — Description of Additional Supplementary Files [file 41467_2024_50011_MOESM3_ESM.pdf]

Description of Additional Supplementary Files

File name: Supplementary Data 1
Description: Label-free quantitative (LFQ) mass spectrometry analysis of FLAG-IP of GTPBP8::FLAG.

File name: Supplementary Data 2
Description: Label-free quantitative (LFQ) mass spectrometry analysis of the GTPBP8-BioID experiment.

File name: Supplementary Data 3
Description: LFQ mass spectrometry analyses of mitoproteome of GTPBP8 knock-out cell lines compared to HEK293.
