# Supplementary material for: GTPBP8 plays a role in mitoribosome formation in human mitochondria
Source: Nat Commun. 2024 Jul 5;15:5664. doi: 10.1038/s41467-024-50011-x (PMC11229512; doi:10.1038/s41467-024-50011-x)
Supplement: Supplementary file 7 — Reporting Summary [file 41467_2024_50011_MOESM7_ESM.pdf]

nature portfolio

Corresponding author(s): Joanna Rorbach

Last updated by author(s): 2024.04.10

Reporting Summary

Nature Portfolio wishes to improve the reproducibility of the work that we publish. This form provides structure for consistency and transparency in reporting. For further information on Nature Portfolio policies, see our Editorial Policies and the Editorial Policy Checklist.

Statistics

For all statistical analyses, confirm that the following items are present in the figure legend, table legend, main text, or Methods section.

n/a	Confirmed	
☐	☒	The exact sample size (*n*) for each experimental group/condition, given as a discrete number and unit of measurement
☐	☒	A statement on whether measurements were taken from distinct samples or whether the same sample was measured repeatedly
☐	☒	The statistical test(s) used AND whether they are one- or two-sided<br>*Only common tests should be described solely by name; describe more complex techniques in the Methods section.*
☒	☐	A description of all covariates tested
☒	☐	A description of any assumptions or corrections, such as tests of normality and adjustment for multiple comparisons
☐	☒	A full description of the statistical parameters including central tendency (e.g. means) or other basic estimates (e.g. regression coefficient) AND variation (e.g. standard deviation) or associated estimates of uncertainty (e.g. confidence intervals)
☐	☒	For null hypothesis testing, the test statistic (e.g. *F*, *t*, *r*) with confidence intervals, effect sizes, degrees of freedom and *P* value noted<br>*Give P values as exact values whenever suitable.*
☒	☐	For Bayesian analysis, information on the choice of priors and Markov chain Monte Carlo settings
☒	☐	For hierarchical and complex designs, identification of the appropriate level for tests and full reporting of outcomes
☒	☐	Estimates of effect sizes (e.g. Cohen's *d*, Pearson's *r*), indicating how they were calculated

Our web collection on statistics for biologists contains articles on many of the points above.

Software and code

Policy information about availability of computer code

Data collection	Peptides were analyzed on an Orbitrap Fusion Tribrid mass spectrometer (Thermo Fisher). Ribosomal footprints were sequenced on an Illumina NextSeq 500. Sequencing of the CLIP libraries was carried out on the Illumina NovaSeq 6000 platform. Cells were counted by EVE Automated Cell Counter (NanoEnTek). Cryo-EM data were collected from vitrified grids using a Krios G3i electron microscope (Thermofisher) operated at 300 kV and equipped with a K3 Bioquantum detector (Gatan). Automated data collection software was used during collection (EPU 2, Thermofisher) which was performed at 165,000× EFTEM SA magnification, yielding a calibrated pixel size of 0.505 Å. 1 sec exposures yielded a total fluency of 48 e-/Å2 in 60 frames, with targeted defocus values -0.3 to -1.1 µm.
Data analysis	Proteomics data were analyzed using MaxQuant, version 1.6.17.0. Differential expression analysis was performed using limma, version 3.34.9 in R, version 3.4.3. Pathway enrichment analysis was performed using WebGestalt.<br>Ribosome profiling sequencing data were analyzed using the MitoRiboSeq analysis pipeline (PMID: 33953394) (open-source software and custom Python and R codes). Pooled sequencing data was split by bcl2fastq conversion software (Illumina). 3'-adapters were trimmed using Cutadapt (DOI: 10.14806/ej.17.l.200). Sequences were aligned using Burrows-Wheeler Aligner (BWA) (DOI: 10.1093/bioinformatics/btp698). A-sites were identified using Plastid (DOI: 10.1186/S12864-016-3278-X).<br>For CLIP data processing Bcl2fastq (v2.20.0), Cutadapt (cutadapt 1.15 with Python 3.6.4), PARpipe (https://github.com/ohlerlab/PARpipe) and Paralyzer were used.<br>Image analysis was performed in ImageJ version 2.0.0 and Image Lab 6.0.1.<br>Statistical analysis was performed using GraphPad Prism version 9.4.0.<br>For cryoEM data, Motion correction, CTF-estimation, Fourier cropping (to 1.02 Å/px), picking and extraction in 512 pixel boxes were performed on the fly using WARP 1.0.9. Cryosparc v4 was used to run 2D classification and further analyses.

For manuscripts utilizing custom algorithms or software that are central to the research but not yet described in published literature, software must be made available to editors and reviewers. We strongly encourage code deposition in a community repository (e.g. GitHub). See the Nature Portfolio guidelines for submitting code & software for further information.

Data

Policy information about availability of data

All manuscripts must include a data availability statement. This statement should provide the following information, where applicable:

- Accession codes, unique identifiers, or web links for publicly available datasets
- A description of any restrictions on data availability
- For clinical datasets or third party data, please ensure that the statement adheres to our policy

Ribosome profiling data are deposited at the GEO under accession number GSE242965. The mass spectrometry data are deposited to the ProteomeXchange Consortium via the PRIDE 46 partner repository with the dataset identifier PXD045264. CLIP data are deposited at the GEO under accession number: GSE256250. Cryo-EM maps were deposited in the Electron Microscopy Data Bank (EMDB). The mt-LSU state 1 and state 2 accession codes are EMD-18460 and EMD-18461, respectively. The mt-SSU state 1, state 2, state 3 and state 4 accession codes are EMD-18438, EMD-18439, EMD-18440 and EMD-18443, respectively.

Research involving human participants, their data, or biological material

Policy information about studies with human participants or human data. See also policy information about sex, gender (identity/presentation), and sexual orientation and race, ethnicity and racism.

Reporting on sex and gender	N/A
Reporting on race, ethnicity, or other socially relevant groupings	N/A
Population characteristics	N/A
Recruitment	N/A
Ethics oversight	N/A

Note that full information on the approval of the study protocol must also be provided in the manuscript.

Field-specific reporting

Please select the one below that is the best fit for your research. If you are not sure, read the appropriate sections before making your selection.

☒ Life sciences ☐ Behavioural & social sciences ☐ Ecological, evolutionary & environmental sciences

For a reference copy of the document with all sections, see nature.com/documents/nr-reporting-summary-flat.pdf

Life sciences study design

All studies must disclose on these points even when the disclosure is negative.

Sample size	Sample sizes were determined based on field standards for obtaining sufficient statistical power (at least 3 biological replicates). Exact sample sizes are depicted in Figure legends.
Data exclusions	No data were excluded from the analysis.
Replication	Two different knockout cell lines for the same gene were analyzed . Biochemical experiments including quantitative mass spectrometry, northern blotting, de novo mitochondrial translation assay, protein steady state levels, OXPHOS activity assay and in vitro translation assay were performed at least three times. All attempts in replication were successful.
Randomization	Positive clones from GTPBP8 knockout generation were randomly chosen. Randomization was not required for this study, as (i) no human or animal subjects were studied, (ii) quantitative data were collected, (iii) there was no danger of confounding independent variables in the experimental design.
Blinding	Blinding was not relevant to this study. For biochemical characterization of cell lines the investigator has to be aware of the sample identity.

Reporting for specific materials, systems and methods

We require information from authors about some types of materials, experimental systems and methods used in many studies. Here, indicate whether each material, system or method listed is relevant to your study. If you are not sure if a list item applies to your research, read the appropriate section before selecting a response.

Materials & experimental systems

n/a	Involved in the study
	☒ Antibodies
	☒ Eukaryotic cell lines
☒	Palaeontology and archaeology
☒	Animals and other organisms
☒	Clinical data
☒	Dual use research of concern
☒	Plants

Methods

n/a	Involved in the study
☒	ChIP-seq
☒	Flow cytometry
☒	MRI-based neuroimaging

Antibodies

Antibodies used

All antibodies used in this study are listed in detail in Table S2.
Anti-FLAG Abcam ab1257
GTPBP8 Prestige Antibodies, Sigma-Aldrich HPA034831
GAPDH Abcam ab8245
SDHA Abcam ab14715
MRPL3/uL3m Prestige Antibodies, Sigma-Aldrich HPA043665
MRPL37/mL37 Prestige Antibodies, Sigma-Aldrich HPA025826
MRPL12/bL12m Prestige Antibodies, Sigma-Aldrich HPA022853
MRPL28/bL28m Prestige Antibodies, Sigma-Aldrich HPA030594
MRPL49/mL49 Proteintech Group 15542-1-AP
MRPL15/uL15m Proteintech Group 18339-1-AP
MRPS16/uS16m Prestige Antibodies, Sigma-Aldrich HPA054538
MRPS37/mS37 (CHCHD1) Invitrogen PA-58635
MRPS15/uS15m Proteintech Group 17006-1
MRPS17/uS17m Proteintech Group 18881-1
MRPS22/mS22 Thermo Fisher 10984-1-AP
RPLP0/uL10 Invitrogen PA5-89335
OXPHOS human WB Abcam ab110411
TOM20 Santa-Cruz sc-11415
MTG1 Prestige Antibodies, Sigma-Aldrich HPA037827
MRM3/RNMTL1 Prestige Antibodies, Sigma-Aldrich HPA023292
RBFA Abcam ab224741
GTPBP10 Prestige Antibodies, Sigma-Aldrich HPA021076
TRMT10C Prestige Antibodies, Sigma-Aldrich HPA036671
HRP secondary rabbit GE Healthcare NA9340V
HRP secondary mouse GE Healthcare NA9310V
HRP secondary goat Santa-Cruz sc-2354

Validation

Antibodies: anti-MRPL3/L37/L12/L28/MTG1/MRM3/GTPBP10/TRMT10C -are Sigma Prestige antibodies validated by the manufacturer (in Western blot using lysates of human cell lines RT-4 and U-251 MG sp-information from the manufacturer website); MRPS16/S37 - validated by the manufacturer (in Western blot using lysates of human cell lines Hela, MCF-7, Raji, in IP experiment from mouse brain); MRPS15/S17/MRPL49/L15 - validated by the PTG manufacturer (in Western blot using lysates of human cell lines Hela, MCF-7, Raji, in IP experiment from mouse brain); CHCHD1 - validated by the manufacturer (in Western blot using lysates of 293 and mouse liver); RPLP0 - validated by the manufacturer (in Western blot using lysates of human and mouse tissues); TOM20 - validated by the manufacturer (in Western blot using lysates of several human cell lines); OXPHOS cocktail human and FLAG - validated by the manufacturer (in Western blot using lysates of human B cell lymphoma lines); RBFA - validated by the manufacturer (in Western blot using lysates of human, mice and rat tissues); SDHA - validated by the manufacturer (in Western blot using lysates of wild-type and SDHA-deficient SH-SYSY cell lines); GAPDH - validated by the manufacturer (in Western blot using lysates of human, mice and rat tissues); GTPBP8 - validated in this study (in Western blot, by comparing wild-type HEK293 line lysate with those of generated GTPBP8-deficient and -overexpressing lines)

Eukaryotic cell lines

Policy information about cell lines and Sex and Gender in Research

Cell line source(s)

Flp-In TREx cell line was purchased from Thermofisher Scientific (catalog number: R78007). GTPBP8::FLAG, GTPBP8-BirA*-HA, mito-BirA*-HA, GTPBP8 KO1 and 2, GTPBP8 RESCUE, mS27::FLAG and AUH -overexpressing cell lines were generated from Flp-In TREx cell line as described in Methods.

Authentication

Generation of GTPBP8 knockout cell lines was validated by sequencing and western blotting (using anti-GTPBP8 antibody). Overexpressing cell lines: GTPBP8::FLAG, GTPBP8 RESCUE (FLAG), mS27::FLAG, GTPBP8-BirA*-HA, mito-BirA*-HA were validated by western blotting (using anti- FLAG and -HA antibodies).

Mycoplasma contamination

Cell line tested negative for mycoplasma contamination.

Commonly misidentified lines<br>(See ICLAC register)	No commonly misidentified cell lines were used in the study.
